# Response to Pegylated Interferon Plus Ribavirin in Patients with Hepatitis C Virus Genotype 6a Infection from Guangdong and Guangxi Province of China

**DOI:** 10.1155/2016/5397407

**Published:** 2016-02-28

**Authors:** Wangxia Tong, Jianyun Zhu, Ning Luo, Xiaohua Yang, Zhiying Lei, Xiaoliang Huang, Zhixin Zhao, Xiaohong Zhang, Zhiliang Gao, Zhonghua Jiang

**Affiliations:** ^1^Ruikang Hospital Affiliated to Guangxi University of Chinese Medicine, Nanning 530000, China; ^2^Department of Infectious Diseases, the Third Affiliated Hospital of Sun Yat-Sen University, Guangzhou 510080, China; ^3^Pingguo County People's Hospital, Guangxi 531400, China; ^4^Department of Emergency, Affiliated Liuzhou Hospital of Guangxi Traditional Chinese Medicine University, Guangxi 545007, China

## Abstract

*Aim.* Our aim is to survey the treatment effect of PEG-IFN plus ribavirin in patients infected with HCV genotype 6a in Guangdong and Guangxi province of China and investigate best course of antiviral treatment for patients with HCV-6a infection.* Methods.* 515 eligible patients received subcutaneous 180 *μ*g PEG-IFN*α*-2a or 1.5 *μ*g/kg PEG-IFN*α*-2b once weekly plus oral ribavirin. Primary outcome was SVR by intention-to-treat analysis. Secondary outcome was RVR, cEVR, ETR, and relapse rate.* Results.* SVR in patients with HCV-6a infection treated for 48 weeks was comparable to that in patients with HCV-2/3 infection (80.9% versus 82.5%, *p* = 0.812) and higher than that in patients with HCV-1b infection (80.9% versus 67.2%, *p* = 0.014). ETR (98.9% versus 90.6%, *p* = 0.016), virological response at month 3 of end-of- treatment (88.8% versus 76.6%, *p* = 0.044), SVR (80.9% versus 65.6%, *p* = 0.032), and virological response at month 12 of end-of-treatment (76.4% versus 60.9%, *p* = 0.04) in patients with HCV-6a infection treated for 48 weeks were higher than those in patients with HCV-6a infection treated for 24 weeks.* Conclusion.* SVR in patients with HCV-6a treated for 48 weeks was comparable to that in patients with HCV-2/3 infection and higher than that in patients with HCV-1b infection; patients with HCV-6a infection treated for 48 weeks had a superior treatment response than patients treated for 24 weeks.

## 1. Introduction

Although the treatment of hepatitis C virus (HCV) has made significant advances with development of nucleotide polymerase inhibitors antivirals currently [1], PEG-IFN and ribaviron-based therapy will still be the mainstay of HCV treatment in China Mainland in the near future, because most new medications may be either unavailable or unaffordable. According to the mass of clinical reports, the therapeutic effect of pegylated interferon (PEG-IFN) and ribavirin is related to various factors, including genotype, age, treatment adherence, and RVR, where genotype is the most important one [2, 3]. HCV is classified into 6 genotypes and more than 90 subtypes and shows diverse patterns of geographic distribution [4, 5]. HCV-1b was still the most widely distributed genotype in China Mainland, followed by 2a, 6a, 3a, and 3b [6, 7]. In accordance with Gu et al. study in 2013 [8], genotype 6a has displaced genotype 2a and became the second genotype in Pearl River Delta China. For antiviral treatment course of PEG-IFN plus ribavirin for CHC patients infected with HCV-6a in China Mainland, the Chinese physicians have often taken the recommended 48-week course adopted by patients infected HCV-1b [9]. It is debatable whether it is suitable for CHC patients infected with HCV-6a in China Mainland. After all, the treatment guideline of 48 weeks is made for patients infected with HCV-1b. According to few clinical report regarding treatment for CHC patients infected with genotype 6 [10, 11], duration of 24 weeks therapy for patients with RVR appeared equally effective as 48 weeks of therapy. And we want to know whether 24 weeks could be standard duration for CHC patients infected with genotype 6a. The data about antiviral therapy for CHC patients in China Mainland are sparse and these experiments are often restricted by the amount of samples and lack of representation [11]. Therefore, future research needs to be based upon more large survey sample in representative regions. The objective of this study is to research the therapeutic effect of PEG-IFN and ribavirin combination for CHC patients with various genotypes in Guangdong and Guangxi province of China and explore the best duration of treatment for CHC patients infected with genotype 6a.

## 2. Objects and Method

### 2.1. Patients

CHC patients with HCV-RNA positive are from the division of infectious diseases at the third affiliated hospital, Sun Yat-Sen University and Hepatology Center of Ruikang Hospital Affiliated to Guangxi University of Chinese Medicine, between December 2012 and June 2015. Routinely, a simple blood test is used to screen the patients for chronic liver disease. The latter refers to the fact that during the past six months or for a longer period ALT (alanine aminotransferase) elevation (>30 U/L) in serum is repeatedly or persistently tested. All eligible patients are enrolled in a registry and created follow-up records. The inclusion criteria included (1) presence of anti-HCV antibody (Roche Diagnostics, Branchburg, New Jersey, USA), (2) detectable serum HCV RNA determined by real-time RT-PCR analysis (COBAS Amplicor HCV Monitor Test, v2.0; Roche Diagnostics, Branchburg, NJ; limit of quantitation 600 IU/mL) for >6 months, (3) HCV genotypes 1b, 6a, and 2/3 determined by nucleotide sequencing of E1 and NS5B region followed by phylogenetic analysis, (4) elevated alanine aminotransferase (ALT) levels 3 times greater than the upper limit of normal, and (5) histological features of chronic inflammation in the past 3 months. The patients were excluded if they had prior antiviral treatment. Further exclusion criteria included (1) anemia (male Hb < 13 g/dL, female Hb < 12 g/dL), (2) neutropenia (neutrophil count < 1500 count/mm^3^), (3) thrombocytopenia (blood platelet count < 70,000 count/mm^3^), (4) coinfection with hepatitis B virus or human immunodeficiency virus, (5) coinfection with HCV nongenotypes 1b, 6a, and 2/3, (6) combined with other serious complications, (7) alcoholism (alcohol intake > 20 g/d), (8) drug addicts, (9) uncontrolled psychiatric or autoimmune disease, and (10) pregnancy and lactation.

### 2.2. Design

The eligible objects was offered prescribed course of treatment according to different HCV genotypes, 48 weeks for genotype 1b, and 24 weeks for genotype 2/3 [3, 12], 48 and 24 weeks for genotype 6a respectively [13]. Patients were treated with either PEG-IFN*α*-2a (180 *μ*g subcutaneously once weekly) or PEG-IFN*α*-2b (1.5 *μ*g/kg/week subcutaneously) in combination with oral ribavirin (a weight-based dose, 15 mg/kg/day). The patients received their first and second PEG-IFN treatment in inpatient clinics for two weeks in order to observe serious side effects. Then, patients are scheduled to outpatient hospital departments for subsequent antiviral treatment and had at least a 6-month follow-up period after the end of treatment.

### 2.3. Follow-Up

No patients had reduced drug doses. Patients were evaluated clinically along with laboratory testing (including complete blood cell count, serum ALT and AST level, serum albumin level, and bilirubin level), at entry, every 4 weeks during the treatment, and every 12 weeks after end-of-treatment, to assess efficacy and safety of treatment. Serum HCV RNA levels were measured at baseline, week 4, week 12, end-of-treatment, and week 24 after end-of-treatment. HCV genotyping was performed at baseline by nucleotide sequencing, genotypes were determined by phylogenic analysis using NS5B and E1 sequences [14]. The assessment of degree of liver fibrosis was done by the noninvasive method of transient elastography (Fibroscan). Based on Lemoine et al. report [15], we selected the cut point of ≥8.7 kpa for ≥F2 stage fibrosis, the cut point of ≥9.5 kpa for ≥F3 stage fibrosis, and the cut point of ≥14.5 kpa for ≥F4 stage fibrosis.

### 2.4. Ethics

The study was approved by hospital ethics committee and all patients provided written informed consent.

### 2.5. Efficacy Assessment

The primary outcome of efficacy was SVR (sustained virologic response), defined as an undetectable serum HCV RNA level at week 24 after treatment. RVR (rapid virologic response) was defined as an undetectable serum HCV RNA level at week 4 of therapy. cEVR (complete early virologic response) was defined as an undetectable serum HCV RNA level at week 12 of therapy. ETR (end-of-treatment response) was defined as an undetectable serum HCV RNA level at end-of-treatment. The patients dropped out of study were considered as achieving a SVR unsuccessfully. Relapse was defined as achieving ETR but becoming positive for HCV RNA after the cessation of treatment. VR 2 W (virological response at week 2 of therapy) is defined as an undetectable serum HCV RNA level at week 2 of therapy. VR 3 M (virological response at month 3 of end-of-treatment) is defined as an undetectable serum HCV RNA level at month 3 of end-of-treatment. VR 12 M (virological response at month 12 of end-of-treatment) is defined as an undetectable serum HCV RNA level at month 12 of end-of-treatment.

### 2.6. Statistical Analysis

Ranked data by Rank-Sum test was used to compare continuous variables if tests normality is observed. Chi-squared tests were used to compare categorical variables across the different genotype groups. The relatedness of potential treatment predictors to SVR was analyzed by univariate and multivariate logistic regression analyses. Statistical significance was defined as a two-side *p* value of 0.05 or less. All analyses were performed using a statistical software package (SPSS, version 16.0; Chicago, IL, USA).

## 3. Results

### 3.1. Patient Characteristics

We collected data from 1342 CHC patients infected with genotype 1b or 2/3 or 6a. The HCV isolates were genotyped by means of a phylogenetic analysis of the E1 and/or NS5B sequences with references representing various genotypes and subtypes. According to inclusion and exclusion criteria, 515 patients were included in final analysis. We identified 153 patients with HCV genotype 6a (64 patients for 24 weeks and 89 patients for 48 weeks), 259 patients with HCV genotype 1b, and 103 patients with HCV genotype 2/3.  Table 1 describes the demographic and baseline laboratory characteristics of patients among different genotype groups (including age, BMI, gender, IDU, blood transfusion, family history of HCC, type of PEG-IFN, ALT, HCV RNA, and cirrhosis). Patients infected with HCV genotype 6a (27.5%) included more intravenous drug users than those infected with other genotype groups (*p* = 0.001). The proportion of patients through blood transfusion in HCV genotype 1b (57.5%) was higher than other genotype groups (*p* < 0.001). Age (*p* = 0.639), gender (*p* = 0.857), BMI (*p* = 0.36), family history of HCC (*p* = 0.302), type of PEG-IFN (*p* = 0.094), and cirrhosis (*p* = 0.118) in four groups were not significant.

### 3.2. Comparison of Virological Response among Patients with HCV-6a Infection Treated for 48 Weeks, HCV-2/3, and HCV-1b Infection

**Figure 1:**
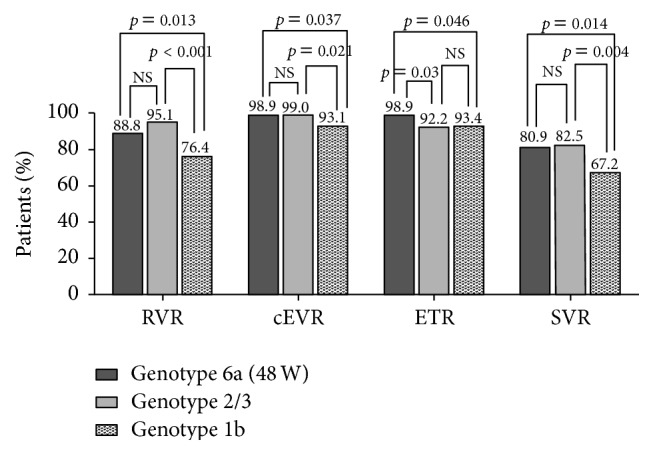
Comparison of the virological response rate to therapy with PEG-IFN plus ribavirin among patients with HCV-6a infection treated for 48 weeks, HCV-2/3, HCV-1b infection. RVR, rapid virologic response; cEVR, complete early virologic response; ETR, end-of-treatment response; SVR, sustained virologic response.

The RVR, cEVR, ETR, and SVR rate were showed in Figure 1. Patients with HCV-6a infection treated for 48 weeks had a higher ETR rate than patients with HCV-2/3 infection (98.9% versus 92.2%, *p* = 0.03). RVR rate (88.8% versus 95.1%, *p* > 0.05), cEVR rate (98.9% versus 99.0%, *p* > 0.05) and SVR rate (80.9% versus 82.5%, *p* > 0.05) in patients with HCV-6a infection treated for 48 weeks were comparable to those in patients with HCV-2/3 infection. RVR rate (88.8% versus 76.4%, *p* = 0.013), cEVR rate (98.9% versus 93.1%, *p* = 0.037), ETR rate (98.9% versus 93.4%, *p* = 0.046), and SVR rate (80.9% versus 67.2%, *p* = 0.014) in patients with HCV-6a infection treated for 48 weeks were higher than those in patients with HCV-1b infection. Although ETR rate in patients with HCV-2/3 infection was comparable to that in patients with HCV-1b infection (*p* = 0.684), RVR rate, cEVR rate, and SVR rate in patients with HCV-2/3 infection were higher than those in patients with HCV-1b infection (*p* < 0.05). The rate of relapse in patients infected with HCV-1b (23.6%) was higher than that in patients infected with HCV-6a treated for 48 weeks (15.3%, *p* = 0.236) and HCV-2/3 (9.2%, *p* = 0.018) during 6-month follow-up after end-of-treatment. The rate of relapse in patients with HCV-6a infection treated for 48 weeks was comparable to that in patients with HCV-2/3 infection (*p* = 0.298).

### 3.3. Comparison of Virological Response between Patients with HCV-6a Infection Treated for 48 Weeks and 24 Weeks

**Figure 2:**
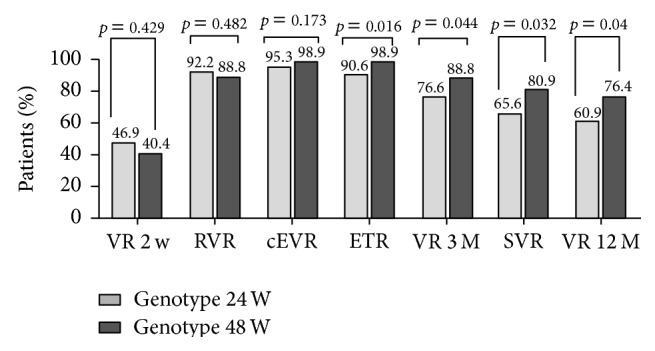
Comparison of the virological response rate between patients with HCV-6a infection treated for 48 weeks and 24 weeks. VR 2 W, virological response at week 2 of therapy; VR 3 M, virological response at month 3 of end-of-treatment; VR 12 M, virological response at month 12 of end-of-treatment. RVR, rapid virologic response; cEVR, complete early virologic response; ETR, end-of-treatment response; SVR, sustained virologic response.

We compared virological response between patients infected with HCV-6a treated for 48 weeks and patients infected with HCV-6a treated for 24 weeks, and RVR, cEVR, ETR, and SVR rate were showed in Figure 2. Virological response at week 2 of therapy (*p* = 0.429), RVR (*p* = 0.482), and cEVR (*p* = 0.173) in patients with HCV-6a infection treated for 48 weeks were similar to those in patients with HCV-6a infection treated for 24 weeks. ETR rate (98.9% versus 90.6%, *p* = 0.016), virological response at month 3 of end-of-treatment (88.8% versus 76.6%, *p* = 0.044), SVR (80.9% versus 65.6%, *p* = 0.032), and virological response at month 12 of end-of-treatment (76.4% versus 60.9%, *p* = 0.04) in patients with HCV-6a infection treated for 48 weeks were higher than those in patients with HCV-6a infection treated for 24 weeks. The rate of relapse and drop-out rate in patients with HCV-6a infection treated for 48 weeks were comparable to those in patients with HCV-6a infection treated for 24 weeks (*p* > 0.05).

**Figure 3:**
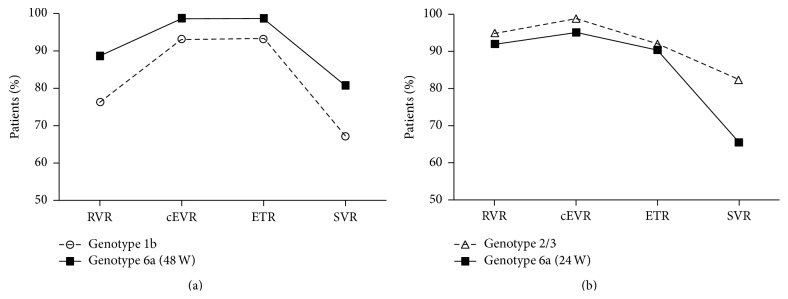
The trend graph of virological response to combined therapy among patients with HCV-1b and HCV-6a-48W (a), HCV-2/3 and HCV-6a-24W (b). RVR, rapid virologic response; cEVR, complete early virologic response; ETR, end-of-treatment response; SVR, sustained virologic response.

The trend graph on virological response to combined therapy among patients with different HCV genotype infections was showed in Figure 3. For patients with HCV-6a infection treated for 48 weeks and patients with HCV-1b infection, virological response rate increased rapidly from week 2 of therapy (RVR) to week 12 of therapy (cEVR), virological response rate was basically flat from week 12 of therapy (cEVR) to end-of-treatment (ETR), and virological response rate was trending downward from end-of-treatment (ETR) to month 6 of end-of-treatment (SVR). For patients with HCV-6a infection treated for 24 weeks and patients with HCV-2/3 infection, virological response rate increased slowly from week 2 of therapy (RVR) to week 12 of therapy (cEVR), virological response rate decreased slowly from week 12 of therapy (cEVR) to end-of-treatment (ETR), and there was no an obvious plateau from cEVR to ETR. Virological response rate was trending downward from end-of-treatment (ETR) to month 6 of end-of-treatment (SVR).

### 3.4. Predictive Factors Associated with SVR in Patients with HCV-6a Infection

Potential predictors of SVR in patients with HCV-6a infection including age, gender, BMI, cirrhosis, ALT level, HCV RNA level, genotypes, cEVR, and RVR were examined. In univariate analysis, age (OR = 4.136, 95% CI: 1.240–13.802, *p* = 0.021), cirrhosis (OR = 7.038, 95% CI: 1.119–44.256, *p* = 0.038), and RVR (OR = 5.798, 95% CI: 2.056–16.354, *p* = 0.001) were associated with SVR. In multivariate analysis to identify predictor of SVR in patients with HCV-6a infection, the independent factor associated with SVR was age (OR = 5.772, 95% CI: 1.153–28.898, *p* = 0.033).

## 4. Discussion

In this study, we researched effect of PEG-IFN plus ribavirin therapy in 515 CHC patients with different genotype in Guangdong and Guangxi province of China. We found that SVR in patients with HCV-6a infection treated for 48 weeks was comparable to that in patients with HCV-2/3 infection and higher than that in patients with HCV-1b infection; patients with HCV-6a infection treated for 48 weeks had a superior treatment response than patients treated for 24 weeks. For patients with HCV-6a infection, age of the subjects was significantly associated with SVR.

This study is the largest population-based retrospective research about therapeutic effects of PEG-IFN plus ribavirin on CHC patients from China Mainland so far and focuses on HCV genotype 6a mainly. The patients (patients infected with HCV-6a mainly) in this study came from the southern coastal region of China Mainland (Guangdong and Guangxi provinces). Guangdong province, home to the Pearl River Delta, is one of the most important trade and financial center in China, Guangxi Zhuang Autonomous Region, located in the southwest coastal region of China, adjoined with Southeast Asian nations. Due to their special geographical situation and the complexity and variety of HCV genotype distribution, the two provinces have the representation in pattern of HCV genotypes in Southern China.

24 weeks of PEG-IFN plus ribavirin therapy were the internationally accepted standard treatment duration for HCV-2/3, and SVR in patients with HCV genotype 2/3 in Asia region was 75%–100%. The rate of SVR in patients with HCV genotype 2/3 (82.5%) in this study falls in line with that in most other reports (75%–100%) [16, 17].

Although ETR was superior in patients with HCV-6a infection treated for 48 weeks versus HCV-2/3 infection (*p* = 0.03). There were no significant differences in RVR, cEVR, and SVR in patients with HCV-6a infection treated for 48 weeks and patients with HCV-2/3 infection (*p* > 0.05). The rate of relapse in patients with HCV-6a infection treated for 48 weeks was comparable to that in patients with HCV-2/3 infection (*p* = 0.298). The results demonstrated that patients with HCV-6a infection treated with PEG-IFN and ribavirin combination treatment for 48 weeks have similar effect as patients with HCV-2/3. And the finding in our study was consistent with the result as Nguyen et al. reported [13].

Our surveys showed that SVR in patients with HCV-1b infection (67.2%) is comparable to what has been reported by other Asian area studies (59%–76) [18–20]. RVR, cEVR, ETR, and SVR were inferior in patients with HCV-1b infection versus patients with HCV-6a treated with 48 weeks (*p* < 0.05). Although ETR in patients with HCV-2/3 infection were no different than that in patients with HCV-1b infection, RVR, cEVR, and SVR in patients with HCV-2/3 infection were higher than those in patients with HCV-1b infection (*p* < 0.05). The rate of relapse in patients with HCV-1b infection was highest. These survey results indicated that patients with HCV-1b infection treated with PEG-IFN and ribavirin combination treatment have worse effect.

As had been reported by Zhou and Thu earlier [10, 11], they believed patients with HCV-6a treated with PEG-IFN and ribavirin combination treatment for 24 weeks have similar effect as patients with HCV-6a treated for 48 weeks. Our results showed that SVR was inferior in patients with HCV-6a infection treated for 24 weeks versus 48 weeks (*p* = 0.032). Virological response at week 2 of therapy (*p* = 0.429), RVR (*p* = 0.482), and cEVR (*p* = 0.173) in patients with HCV-6a infection treated for 48 weeks were similar to those in patients with HCV-6a infection treated for 24 weeks, so there was no significant difference between the two groups above. But ETR rate (*p* = 0.016), virological response at month 3 of end-of-treatment (*p* = 0.044), SVR (*p* = 0.032), and virological response at month 12 of end-of-treatment (*p* = 0.04) in patients with HCV-6a infection treated for 48 weeks were higher than those in patients with HCV-6a infection treated for 24 weeks, and there was significant difference between the two groups above. What drives the differences? According to our analysis, it is the course of treatment. Time span was 12 weeks from week 12 of therapy (EVR) to end-of-treatment (ETR) for patients with HCV-6a infection treated for 24 weeks and 36 weeks for patients with HCV-6a infection treated for 48 weeks. We can think that the difference in virologic response between two treatment groups appeared to be due to difference in time length of therapy. The possibility of difference in the demographic and baseline laboratory characteristics and drop-out rate was ruled out, because there was no significant difference in the characteristic between two groups.

In accordance with the trend graph of virological response to combined therapy among patients with different HCV genotype infections, a line of rise first then fall was presented on HCV-6a (48 weeks) group and HCV-1b (48 weeks) group, there was an obvious plateau in the middle of the line, and the trend graph was trapezoid-shaped. We think the excellent effect in treatment can be maintained by longer-lasting therapy and manifested as the plateau. A trend graph of rise first then fall was also presented on HCV-6a (24 weeks) group and HCV-2/3 (24 weeks) group, but there was not an obvious plateau. Virological response at week 12 of therapy (cEVR) was the highest point. We can consider that short-term combined treatment (24 weeks) was inferior to longer-term treatment (48 weeks) for patients with HCV-6a infected.

The weakness in the study was retrospective design, and there was sample selection bias in patients with HCV-6a infection treated for 24 weeks and 48 weeks. What is important is that the sample size was small for patients with HCV-6a infection. Future researches need to be based upon more large survey HCV-6a samples and randomized controlled trials.

## 5. Conclusion

SVR in patients with HCV-6a infection treated for 48 weeks was comparable to that in patients with HCV-2/3 infection and higher than that in patients with HCV-1b infection; patients with HCV-6a infection treated for 48 weeks had a superior treatment response than patients treated for 24 weeks. For patients with HCV-6a infection, age of the subjects was significantly associated with SVR.

## Figures and Tables

**Table 1:** The baseline characteristics of patients with different HCV genotype infection.

Characteristics	HCV-6a (*n* = 153)		HCV-2/3 (*n* = 103)	HCV-1b (*n* = 259)	*p* value^*∗*^
	(24 w, *n* = 64)	(48 w, *n* = 89)			
Age (years)	34.2 ± 11.6	36.6 ± 10.6	36.5 ± 14.06	36.7 ± 13.03	0.639
BMI	22.0 ± 4.0	22.1 ± 3.9	22.1 ± 2.62	22.4 ± 3.93	0.36
Male (*n*)	40 (62.5%)	59 (63.3%)	62 (60.2%)	163 (62.9%)	0.857
IDU (*n*)^†^	17 (26.6%)	25 (28.1%)	14 (13.6%)	24 (9.3%)	<0.001
Blood transfusion (*n*)	15 (23.4%)	21 (23.6%)	37 (35.9%)	149 (57.5%)	<0.001
Family history of HCC (*n*)^†^	4 (6.2%)	6 (6.7%)	4 (3.9%)	7 (2.7%)	0.302
PEG-IFN*α*-2a (*n*)	50 (78.1%)	67 (75.3%)	85 (82.5%)	223 (86.1%)	0.094
ALT (U/L)	82.9 ± 8.1	80.0 ± 6.9	87.9 ± 3.0	78.1 ± 9.0	0.955
HCV RNA (_log_IU/mL)	6.1 ± 1.0	6.2 ± 1.1	5.6 ± 1.1	6.4 ± 0.9	0.106
Cirrhosis (*n*)	6 (9.4%)	5 (5.6%)	9 (8.7%)	37 (14.3%)	0.118

BMI, body mass index; ^†^IDU, intravenous drug use; HCC, hepatocellular carcinoma; ALT, alanine aminotransferase; HCV RNA, hepatitis C virus ribonucleic acid; cirrhosis, the cut point of ≥14.5 kpa for ≥F4 stage fibrosis; ^*∗*^ *p* value comparing the different genotype groups.

## Abbreviations

PEG-IFN: Pegylated interferon
HCV: Hepatitis C virus
SVR: Sustained virologic response
RVR: Rapid virologic response
cEVR: Complete early virologic response
ETR: End-of-treatment response
CHC: Chronic hepatitis C
HCV-RNA: Hepatitis C virus ribonucleic acid
ALT: Alanine aminotransferase
RT-PCR: Reverse transcriptase-polymerase chain reaction
BMI: Body mass index
HCC: Hepatocellular carcinoma.
